# Control of Vascular Risk Factors and Response to Stroke Symptoms in Argentina During the COVID-19 Quarantine. The SIFHON-COVID Population Survey

**DOI:** 10.3389/fneur.2022.826061

**Published:** 2022-04-11

**Authors:** María S. Rodríguez Pérez, Julieta S. Rosales, Daiana E. Dossi, Sebastián F. Ameriso

**Affiliations:** Fundación Para la Lucha Contra las Enfermedades Neurológicas de la Infancia (FLENI), Buenos Aires, Argentina

**Keywords:** stroke, COVID-19, vascular risk factor, isolation, management

## Abstract

**Background and Objectives:**

In preparation for the suspected influx of COVID-19 patients, many healthcare systems reduced or discontinued provision of “non-urgent” care. This decision had potential impact on stroke prevention and management. We conducted a large population survey to assess the effect of mandatory social isolation on routine health controls, emergency consultations and other stroke care-related behaviors of the population during the pandemic.

**Methods:**

We distributed multiple-choice anonymous questionnaires through the institutional email database and through the email database of clients of a beverage delivery company. Most respondents resided in the metropolitan area of Buenos Aires. This is the area where the infection rates were highest and restriction measures were hardest. The survey assessed demographic characteristics and actual and potential behaviors of people regarding medical checkups, risk factors control, medication provision and response to onset of symptoms consistent with stroke or TIA. Surveys were sent during May 2020, the strictest period of the quarantine in Argentina.

**Results:**

A total of 10,303 questionnaires were completed. Thirty-seven percent of the respondents were older than 60 years, 74% were women and 16% lived alone. Vascular risk factors were present in 39% of the individuals. Seventy-six percent did not continue with their regular medical checkups during the mandatory social and preventive isolation, 21% had difficulty obtaining medical prescriptions and only 38% considered that health institutions had implemented reliable safety measures to avoid exposure to COVID-19. When asked about response in case of onset of stroke symptoms, 9% would not consult given the context of the pandemic. Six percent reported having had symptoms consistent with stroke or TIA but only 35% went to a hospital. The vast majority of the respondents said they were awaiting for the end of the quarantine to resume their usual medical care.

**Conclusions:**

The implementation of a quarantine may have some serious adverse effects on the prevention, diagnosis and treatment of stroke. These undesirable aspects should be taken into consideration in the planning, communication and implementation of health policies.

## Introduction

A new strain of a highly contagious coronavirus (SARS-CoV-2) emerged in late 2019 in China and spread to most of the world in early 2020 (1, 2). During the next several months most countries adopted population isolation measures of different intensities and duration. A quarantine is a state of isolation or restricted access instituted as a safety measure. On March 20th, 2020, with 128 confirmed cases and 3 deaths (3), Argentina imposed a nationwide lockdown that was among the strictest in the world. During the “social, preventive and compulsory isolation,” people had to remain in their usual residences or in the residence in which they were at that moment. People were not allowed to travel to their places of work and move along routes, roads and public spaces. Only minimum and essential trips to stock up on cleaning supplies, medicines and food were allowed. There were permanent controls on routes, roads and public spaces. Cultural, recreational, sporting and religious events were banned. Shopping centers, restaurants, wholesale and retail establishments closed (4). Health institutions also took steps to adapt to the pandemic. Most of them established safety and protection measures against the coronavirus allowing entry to the institution exclusively with face masks, requesting a signed statement of absence of symptoms consistent with COVID, close contact with COVID index cases and/or recent trips. It was also mandatory to measure the temperature of patients and professionals upon entering the institution. A dual circulation pathway was established for doctors and patients. In outpatient consultations, only one companion was allowed per patient and crowding of patients in waiting rooms was avoided (5). Some of these strict measures were lifted on May 10th but many were still in effect by September 3rd (6).

Departure from routine life, voluntary or reactionary, caused by any major human event or natural calamity may lead to a ripple effect affecting access to resources and human behavior, especially healthcare seeking behavior. The effect of the COVID-19 pandemic on medical care for conditions other than COVID-19 has been difficult to quantify. Patients may potentially be at higher risk because of late or no consultation (7). The stay-at-home recommendation may lead to increased social isolation, fewer potential witnesses for stroke symptoms onset and hence a reduction in the likelihood of recognition of mild stroke signs and symptoms. Anxiety and fear of contracting the infection in healthcare environments, along with assumptions that hospitals are overwhelmed with COVID-19 patients, may lead to a tendency among certain groups of patients to not seek advanced care and therefore stay at home. The information regarding population behavior around the world in the context of pandemic and isolation is scant. The American College of Emergency Physicians conducted an online 2-day survey in April 2020, obtaining 2201 responses. Four in five adults reported they were concerned about contracting COVID-19 from another patient or visitor if they needed to go to the emergency room and nearly a third of adults (29%) had actively delayed or avoided seeking medical care due to concerns about contracting the disease. When considering a visit to the emergency department, a strong majority (73%) were concerned about overstressing the health care system (8). Furthermore, the CorCOVID LATAM Study (9), a population survey in 13 countries of Latin America addressing the impact of the pandemic on nonInfected cardiometabolic patients with 4,216 responses, showed that 46.4% of patients did not have contact with a healthcare provider, 31.5% reported access barriers to treatments and 17% discontinued some medication.

The SIFHON program was launched in 2015 using household surveys as a research method. The first work was published in 2019, with over 12,700 responses obtained from 12 provinces in Argentina, regarding stroke awareness (10). That same year, SIFHON 2 was carried out, this time in a digital version, to assess the degree of knowledge of the population about cerebrovascular disease, prevalence of the main risk factors and healthy habits. We have also carried out joint work with another population study project (EstEPA) that our team continues to develop to assess knowledge of stroke in the study cohort (11). Numerous branches of the project are ongoing both nationally and internationally in Latin America with the aim of investigating behaviors and knowledge the population regarding stroke.

As part of our SIFHON program of population surveys in stroke we undertook a large study to determine the behavior of the population in the context of the quarantine with respect to stroke prevention and treatment in Argentina.

## Materials and Methods

**Figure 1:**
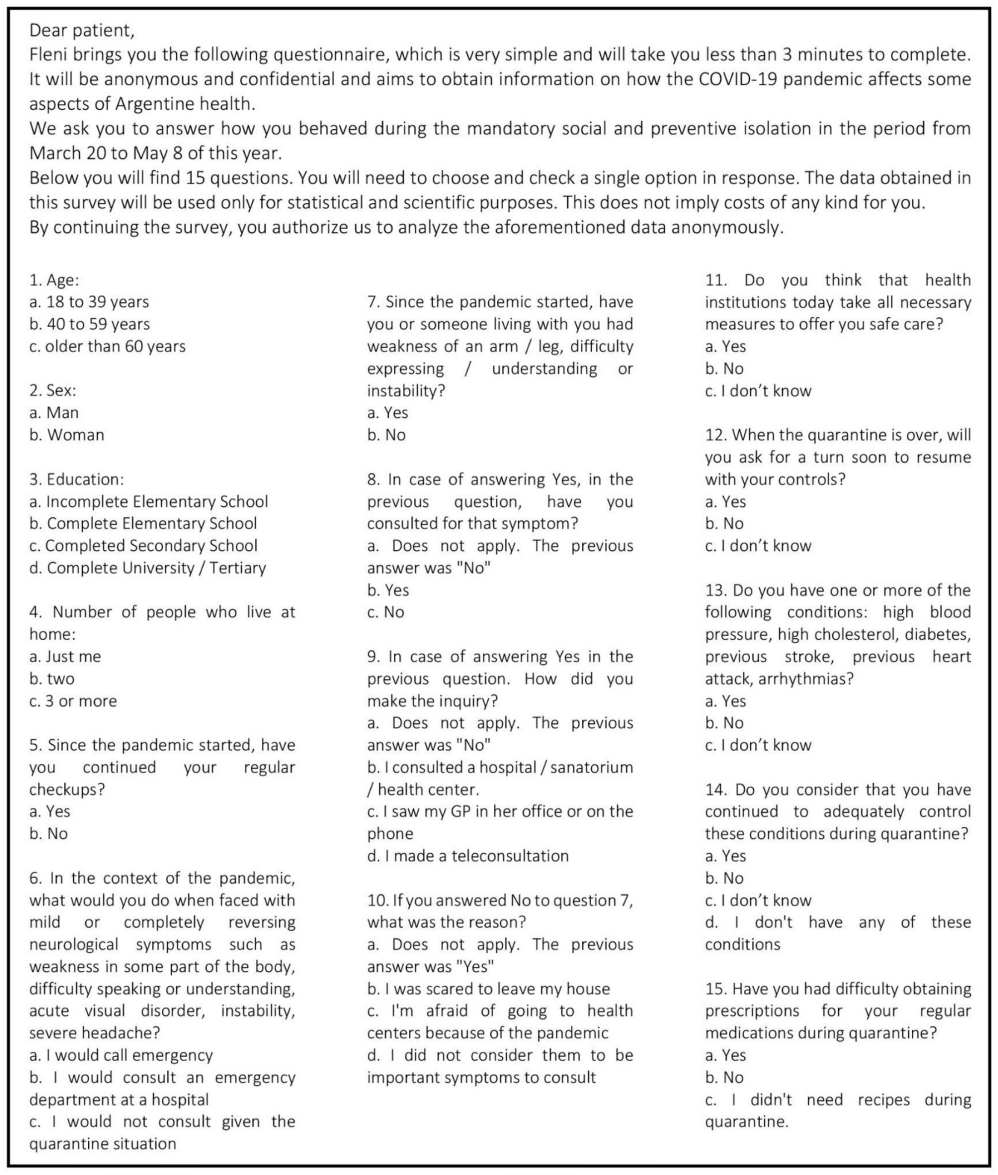
Model of questionnaire used for this survey (translated to English language).

We distributed an anonymous online survey, with 15 multiple-choice close-ended questions (Figure 1) evaluating age, gender, educational level, number of people in the household and presence of vascular risk factors (VRF). They were interrogated for the presence of hypertension, elevated cholesterol, diabetes, previous stroke or heart attack, and arrhythmias. We also asked if the subject had continued his/her regular medical controls, if he/she, considered that medical institutions were safe in terms of risk of contagious of COVID-19 and if they had had difficulties obtaining medical prescriptions. We also interrogated about conduct in case of potential or actual stroke symptoms. The study was reviewed and approved by the institutional ethics committee. All participants signed an informed consent.

The questionnaire was distributed through 2 e-mail databases: Fleni database (mostly former or present patients and benefactors) and the database of regular customers of a beverage delivery company. By using these two databases we achieved a mix of former patients with a need for regular medical check-ups and subjects from the general population with no predetermined needs. The majority of respondents lived in the metropolitan area of Buenos Aires. This is the region where the infection rates were higher and restriction measures were hardest. The survey was sent during the strictest time of the quarantine in Argentina. Questionnaires were circulated in early May and responses were received during 12 consecutive days.

The percentages of responses for each question were calculated for the entire group and for several subgroups according to age, gender and presence of VRF. For statistical analysis, categorical variables are presented as frequencies and percentages. Normal distribution was checked using Shapiro-Wilk test. For comparisons between groups we used chi square test for categorical variables and *t*-test for continuous variables. Differences were considered statistically significant for *p* < 0.05.

## Results

Demographic data is provided in Table 1. A total of 10,303 questionnaires were completed. The results were analyzed and sub-analyses were also performed according to the entire population, presence of VRF, age older than 60 years, people living alone and perceptions about safety of medical institutions.

**Table 1:** Demographic data and presence of vascular risk factors.

**Total 10,303**	**n**	**%**
**Age**		
18–39 years	2,151	21
40–59 years	4,301	42
> 60 years	3,837	37
N/R	14	-
**Gender**		
Female	7,629	74
Male	2,661	26
N/R	13	-
**Cohabitants**		
1	1,628	16
2	3,761	37
3 or more	4,902	48
N/R	12	-
**Vascular risk factors**		
Yes	4,048	39
No	5,939	58
Don't know	299	3
N/R	17	-

*N/R, no response*.

Thirty-seven percent of the respondents were older than 60 years, 74% were women and 16% lived alone. VRF were present in 39%. Behavior of the population during the pandemic is described in Table 2. Seventy-six percent did not continue with regular medical checkups during the quarantine. This was similar for all ages, gender and for subjects with or without VRF. Only 38% of the surveyed considered that health institutions were adopting all the necessary measures to offer safe care (Table 2).

**Table 2:** Adherence to medical controls, perception of safety measures of health institutions and behavior of population regarding stroke symptoms.

**Total 10,303**	**n**	**%**
**Continue with regular medical checkups**		
Yes	2,502	24
No	7,801	76
**Safety measures at hospitals**		
Yes	3,894	38
No	1,373	13
Don't know	5,019	49
N/R	17	-
**Medical prescriptions**		
Need of medical prescriptions	7,319	71
Difficulty obtaining medical prescriptions	1,536	21
**Actions to be taken in the hypothetical case of onset of symptoms consistent with stroke**		
Call emergency	3,014	29
Go to ER	6,358	62
Would not consult	914	9
**Appearance of stroke symptoms during isolation**		
Yes	592	6
No	9,697	94
N/R	14	-
**If yes, did you make a consultation?**		
Yes	244	42
No	348	58
**If consulted, what was the mechanism?**		
Hospital/Health center	85	35
Physicians in office or by phone	100	41
Teleconsultation	56	23
N/R	3	1
**If you did not consult, what was the reason?**		
Scared to leave my house	30	9
Afraid of going to health centers	98	28
Did not consider symptoms to be important	182	52
N/R	38	11

*N/R, no response, respondents who left blank this question or marked incorrectly*.

When asked about actions taken in case of onset of symptoms consistent with stroke, 9% answered that they would not consult given the pandemic. Five hundred and ninety-two subjects (6%) reported having had symptoms indicative of stroke or TIA. Of them 56% did not consult. Approximately half did not consider that symptoms were important, one-third were afraid of going to health centers or leaving their homes. Of those who consulted 35% went to the hospital, 41% talked by the phone with their primary physician and 23% made a teleconsultation. Analyzing the subgroup of those who continued with the usual medical consultations, the percentage of symptoms consistent with stroke was lower (3%) and they consulted more often (68%) compared to subjects that did not continue with their usual consultations who referred symptoms in 6.5% and consulted in 37% of cases (*p* < 0.00001). Lack of consultation was higher in respondents who did not consider that health institutions were taking adequate prevention measures compared to those who did, 38 vs. 62%, px. Within this subgroup, those who presented symptoms consistent with stroke, 60% did not consult vs 41% of consultation in subjects who trusted the institutions and presented symptoms (*p* < 0.001).

Seven thousand three hundred nineteen subjects referred need for medical prescriptions (71%). Of these, 1,536 (21%) had difficulty obtaining them. This problem was more evident among subjects older than 60 years. The vast majority were waiting for the end of the quarantine to resume their usual medical check-ups. As expected, subjects from Fleni's database tended to be older, had more often VRF and more often referred symptoms consistent with strokes. Table 3 shows both study cohorts (medical institution and general population). There were no significant differences between both groups suggesting that some of the deleterious effects of isolation applied to both subjects already under regular medical follow up as well as those from the general population.

**Table 3:** Demographic data, vascular risk factors and behavior of the two cohorts included in the study.

	**Subjects from medical institution (*n* = 6,189)**		**Subjects from the general population (*n* = 4,114)**	
	**n**	**%**	**n**	**%**
**Age**				
18–39 years	965	16	1,186	29
40–59 years	2,435	39	1,866	45
> 60 years	2,775	45	1,062	26
N/R	14	-	-	-
**Gender**				
Female	7,629	74	3,045	74
Male	2,661	26	1,069	26
N/R	13	-	-	-
**Vascular risk factors**				
Yes	2,747	44	1,301	32
No	3,254	53	2,685	65
Don't know	171	3	128	3
N/R	17	-	-	-
**Continued with medical check-ups**				
Yes	1,453	24	1,049	25
No	4,722	76	3,065	75
N/R	14	-	-	-
**Safety measures al hospitals**				
Yes	2,284	37	1,610	39
No	819	13	554	13
Don't know	3,069	50	1,950	47
N/R	17	-	-	-
**Difficulty obtaining medical prescriptions**				
Need of medical prescriptions	4,743	77	2,576	63
Difficulty obtaining medical prescriptions	985	21	551	21
**Actions taken in the hypothetical case of onset of symptoms consistent with stroke**				
Call emergency	1,983	32	1,031	25
Go to ER	3,574	58	2,784	68
Would not consult	615	10	299	7
N/R	17	-	-	-
**Appearance of stroke symptoms during isolation**				
Yes	439	7	153	4
No	5,736	93	3,961	96
N/R	14	-	-	-
**If yes, did you make a consultation? (*n* = 439/153)**				
Yes	182	41	62	41
No	242	55	89	58
N/R	15	3	2	1
**If consulted, what was the mechanism? (*n* = 182/62)**				
Hospital/Health center	55	30	30	48
Physicians in office or by phone	84	46	16	26
Teleconsultation	42	23	14	23
N/R	1	1	2	3
**If you did not consult, what was the reason? (*n* = 242/89)**				
Scared to leave my house	25	10	5	6
Afraid of going to health centers	77	32	21	24
Did not consider symptoms to be important	128	53	54	61
N/R	12	5	9	10

*Subjects from medical institution were those who had been seen at Fleni. Subjects from the general population were clients of the beverage delivery company. N/R, no response, respondents who left blank this question or marked incorrectly*.

## Discussion

Isolation measures due to the COVID-19 pandemic may be detrimental to patients with need for regular medical controls and/or medical emergencies such as stroke. We report that 76% of the population did not continue with their medical controls during the compulsory social and preventive isolation period. Only 38% of the surveyed considered that health institutions had implemented all the necessary measures to warrant safe care. Also, 6% admitted having had symptoms consistent with stroke, however only 42% of them consulted in a medical institution. Twenty-one percent had difficulty getting medical prescriptions and the vast majority were waiting for the end of quarantine to resume their usual medical check-ups.

Presence of baseline poor knowledge of basic stroke facts may have affected some of the responses (8). More than half of the respondents who had symptoms consistent with stroke did not consider them to be important. Thus, the combination of barriers to the access to medical care with poor population knowledge may have potential serious health consequences.

A decrease in stroke hospital admissions, stroke code activations, intravenous thrombolysis and thrombectomies in Europe and USA during the pandemic has already been reported (12–18). During this period stroke admissions and outpatient cerebrovascular consultations dropped drastically at our institution (5). However, data is scant regarding the impact of mobility and isolation restriction measures on regular controls or VRF management and response in case of stroke symptoms. This may have long term consequences not reflected by current statistics. The 2-day survey conducted by the American College of Emergency Physicians showed that the main fear of the population was to be infected with COVID-19. This fear was not only related to the time spent in the medical waiting room but also to the possibility of being in contact with a physician or another patient or companion in the emergency room (8).

Since the implementation of the preventive and compulsory social isolation in Argentina, consultation and hospitalization rates for acute cerebrovascular events have fallen when compared to the same period in 2019 (5, 19). People were reluctant to go to a hospital during the COVID-19 outbreak, especially those who believed that hospitals were not taking the appropriate safety measures (19). In our stroke center, compared to the same 5-month period in 2019, there was a significant decrease in the number of hospitalizations for total ischemic events and transient ischemic attacks. Access to intravenous fibrinolysis and mechanical thrombectomy remained stable, but with prolonged door-to-needle time (5).

Our survey adds information regarding population beliefs and conduct during the pandemic. It confirms the fear of getting infected when attending a health center. The massive communication campaign enforcing the quarantine combined with the lack of confidence about safety at the institutions was reflected in the drop in regular medical check-ups and assistance to health institutions in case of stroke symptoms. In addition, many subjects reported difficulties obtaining prescriptions. The behavior of the population reflected in this questionnaire may be the consequence of fear of getting infected with COVID-19 plus a non-desired effect of the strong “stay at home” message. To the best of our knowledge this is the first study to analyze the influence of COVID-19 pandemic in population behavior regarding management of stroke risk factors and response to stroke symptoms in Latin America. This behavior may have potentially deleterious long-term consequences.

The slogan and hashtag “stay at home” was very effective in reducing population mobility and gathering and controlling the massive influx of patients without clear criteria to emergency departments. It also helped to organize the public and private health system to confront the pandemic. However, our findings emphasize another important message to communicate to the population: “health care should not stay at home” and patients, especially those with VRF, should be strongly recommended to continue with their controls. A paradigm switch from the original “stay at home” message is necessary, encouraging regular check-ups and early consultation at the onset of neurological symptoms consistent with stroke. Furthermore, it is crucial to create new pathways and assistance protocols for patients with non-COVID conditions to restore trust in health institutions.

Our study has some limitations. The survey was self-reported and we designed a closed ended questionnaire. The methodology used may introduce certain selection biases as we considered only data from subjects who responded the questionnaires. Furthermore, the characteristics of the survey did not allow us to control certain aspects of information like self-reporting and interpretation of some health issues. As the pandemic continues, the care systems may adapt and tendencies might change. Furthermore, our results might not be representative of other countries or regions with different restrictions, stroke care protocols and geographical particularities. Besides the Cor COVID LATAM study (9) there are no regional studies on population response to the pandemic.

In conclusion, there are substantial collateral adverse effects of isolation measures during the pandemic concerning the care for other acute and chronic severe conditions, such as stroke. The quarantine resulted in a drop in consultations for regular medical checkups and serious vascular diseases. Many people underestimated neurological symptoms and failed to consult, likely due to strict isolation and lack of confidence in appropriate safety measures at hospitals. Increased public awareness and corrective measures are needed to mitigate the deleterious effects of the COVID-19 outbreak on stroke care.

## Data Availability Statement

The original contributions presented in the study are included in the article/supplementary material, further inquiries can be directed to the corresponding author.

## Ethics Statement

The studies involving human participants were reviewed and approved by the Ethics Committee of Fundación Para la Lucha Contra las Enfermedades Neurológicas de la Infancia (FLENI). Written informed consent to participate in this study was provided by the patients/participants or patient/participants legal guardian/next of kin.

## Author Contributions

MR conceived the original idea, processed the experimental data, performed the analysis, drafted the manuscript and designed the figures. JR and DD verified analytical data. SA encouraged MR, DD, and JR to investigate this topic, supervised the project and the findings of this work. All authors discussed the results and contributed to the final manuscript.

## Conflict of Interest

The authors declare that the research was conducted in the absence of any commercial or financial relationships that could be construed as a potential conflict of interest.

## Publisher's Note

All claims expressed in this article are solely those of the authors and do not necessarily represent those of their affiliated organizations, or those of the publisher, the editors and the reviewers. Any product that may be evaluated in this article, or claim that may be made by its manufacturer, is not guaranteed or endorsed by the publisher.

## References

[1] Pneumonia of unknown cause–China. W World Health Organization. Emergencies Preparedness, Response. (2021). Available online at: https://www.who.int/csr/don/05-january-2020-pneumonia-of-unkown-cause-china/en/ (accessed on February 7, 2021).

[2] Guan W Ni Z Hu Y Liang W Ou C He J. Clinical characteristics of coronavirus disease 2019 in China. N Engl J Med. (2020) 382:1708–20. 10.1056/NEJMoa2002032 32109013 PMC7092819

[3] Reporte diario Nro 15. Situación de COVID-19 en Argentina. Nuevo coronavirus COVID-19. Informe Diario. Ministerio de Salud (2021). Available nline at: https://www.argentina.gob.ar/sites/default/files/19-03-20-reporte-diario-covid-19.pdf (accessed on February 7, 2021).

[4] Aislamiento Social Preventivo y Obligatorio. Decreto 297/2020. Argentina Presidencia. Boletín Oficial de la República Argentina. Legislación y Avisos Oficiales. Primera sección (2021). Available online at: https://www.boletinoficial.gob.ar/detalleAviso/primera/227042/20200320 (accessed on February 7, 2021).

[5] Rosales JS Rodriguez-Perez MS Ameriso SF. Efecto de la pandemia COVID-19 y la cuarentena en el número de consultas, subtipos y tratamiento del accidente cerebrovascular en un centro neurológico de Argentina [Effect of the COVID-19 pandemic and preventive social isolation measures on the number of outpatient visits, hospitalizations and treatment of cerebrovascular accident in a neurological center in Argentina]. Medicina Buenos Aires. (2020) 80(Suppl. 6), 65–70. 33481735

[6] Aislamiento Social Preventivo y Obligatorio. Decreto 459/2020. Argentina Presidencia. Boletín Oficial de la República Argentina. Legislación y Avisos Oficiales. Primera sección. Available online at: https://www.boletinoficial.gob.ar/detalleAviso/primera/228958/20200511 (accessed on February 7, 2021).

[7] Pujol Lereis VA Flores A Barboza MA Abanto-Argomedo C Ameriso SF. COVID-19 lockdown effects on acute stroke care in Latin America. J Stroke Cerebrovasc Dis. (2021) 30:105985. 10.1016/j.jstrokecerebrovasdis.2021.105985 34284323 PMC9186152

[8] Morning Consult. COVID-19. American College of Emergency Physicians. April (2020). Available online at: https://www.emergencyphysicians.org/globalassets/emphysicians/all-pdfs/acep-mc-covid19-april-poll-analysis.pdf

[9] Lopez Santi R Márquez MF Piskorz D Saldarriaga C Lorenzatti A. Ambulatory patients with cardiometabolic disease and without evidence of COVID-19 during the pandemic. The CorCOVID LATAM study. Glob Heart. (2021) 16:15. 10.5334/gh.932 33833939 PMC7894376

[10] Dossi DE Hawkes MA Pujol Lereis VA Povedano GP Ameriso SF et al. A population-based survey of stroke knowledge in Argentina: the SIFHON study. Neuroepidemiology. (2019) 53:32–40. 10.1159/000497413 30986784

[11] Hawkes MA Gomez-Schneider MM Dossi DE Melcon MO Ameriso SF. Stroke knowledge in the EstEPA project, a population-based study. J Stroke Cerebrovasc Dis. (2021) 30:105471. 10.1016/j.jstrokecerebrovasdis.2020.105471 33242783

[12] Markus H Martins S. COVID-19 and stroke–understanding the relationship and adapting services. A global World Stroke Organization. Int J Stroke. (2021) 16:241–7. 10.1177/17474930211005373 33709834 PMC8044614

[13] Bersano A Bornstein N. Stroke Care at the Time of COVID-19 Outbreak. European Academy of Neurology (2020). Available online at: https://www.eanpages.org/2020/04/17/stroke-care-at-the-time-of-covid-19-outbreak/ (accessed on December 20, 2020).

[14] Markus HS. Express: COVID-19 and stroke – a global World Stroke Organization perspective. Int J Stroke. (2020) 15:361–4. 10.1177/1747493020923472 32310017 PMC11927026

[15] Rudilosso S Chamorro A Vera V Vargas M Renú A Llull L. Acute stroke care is at risk in the era of COVID-19. Stroke. (2020) 51:1991–5. 10.1161/STROKEAHA.120.030329 32438895 PMC7258755

[16] Montaner J Barragán-Prieto A Pérez-Sánchez S Escudero-Martínez I Moniche F Sánchez-Miura JA. Break in the stroke chain of survival due to COVID-19. Stroke. (2020) 51:2307–14. 10.1161/STROKEAHA.120.030106 32466738 PMC7282408

[17] Pop R Quenardelle V Hasiu A Hasiu A Mihoc D Sellal F. Impact of the Covid-19 outbreak on acute stroke pathways–insights from the Alsace region in France. Eur J Neurol. (2020) 27:1783–7. 10.1111/ene.14316 32399995 PMC7273043

[18] Aguiar de Sousa D Sandset EC Elkind M. The curious case of the missing strokes during the COVID-19 pandemic. Stroke. (2020) 51:1921–3. 10.1161/STROKEAHA.120.030792 32466737 PMC7282410

[19] Calandri I Hawkes MA Marrodan M Ameriso SF Correale J Allegri RF. The impact of an early strict nationwide lockdown on the pattern of consultation for neurological diseases. J Neurol Sci. (2020) 418:117084. 10.1016/j.jns.2020.117084 32818813 PMC7422811

